# Is Syringocystadenoma Papilliferum Incidental in This Verrucous Carcinoma?

**DOI:** 10.1155/2019/1783758

**Published:** 2019-10-22

**Authors:** Tyler Long, Brian Bonomo, Sydney Shearer, William Welton, Ralph Massullo, George Gibbons

**Affiliations:** ^1^Lake Erie College of Osteopathic Medicine, 5000 Lakewood Ranch Blvd, Bradenton, FL 34211, USA; ^2^Suncoast Dermatology, Lecanto, FL, USA; ^3^Medical Director—Dermpath Diagnostics Bay Area of Dermatopathology, Tampa, FL, USA

## Abstract

This case report presents a case in which a collision tumor consisting of three separate pathological entities—a verrucous carcinoma (VC), syringocystadenoma papilliferum (SCAP), and a basal cell carcinoma (BCC). The presentation of this collision tumor is unexpected. It presented as an exophytic mass on the scalp. While collisions of SCAP and VC are present in the literature, this case included an additional pathologic entity. The association of these entities and the unreported location of the lesion may provide some further insight as to the etiology of VC.

## 1. Introduction

We report a case of a collision tumor consisting of a syringocystadenoma papilliferum, nodular-type basal cell carcinoma, and a verrucous carcinoma. These pathologic entities were each represented in a single 6.0 cm lesion. We raise the question, was the SCAP an “incidental” finding or an initiating factor?

Syringocystadenoma papilliferum (SCAP) is a benign adnexal tumor that is most commonly found in females at birth or in early childhood [1]. On rare occasions SCAP will transform to a basal cell carcinoma in 10% of cases [2]. There have been many locations and clinical variations documented, but the majority of them occur on the scalp [3]—as we see in this case.

Verrucous carcinoma (VC) is a clinicopathologic variant of squamous cell carcinoma that is often named according to the location of the lesion. The most common lesions are found on the genitalia, the soles of the feet, and on the oral mucosa. While human papilloma virus (HPV) is often suspected, the etiology of VC is diverse and uncertain [4].

The coexistence of these three diverse dermatological tumors represents a rare association of SCAP and VC; furthermore, it represents a very unlikely location of VC. This association may provide further understanding of the etiology of cutaneous VC.

## 2. Case Report

**Figure 1:**
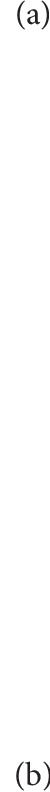
Posterior cranium exhibiting a large exophytic mass.

A 64-year-old male presented to an outpatient dermatologist with a five-year history of a growing mass on his left occipital scalp. The mass has grown consistently larger during this time but delayed seeking help due to financial issues. The patient denied any pain, fluid secretion from the lesion, or constitutional symptoms. He denied any history of lesions prior to this occipital mass and reports being healthy. On physical examination, a 6.0 cm exophytic mass is found on the left occipital scalp (Figure 1).

**Figure 2:**
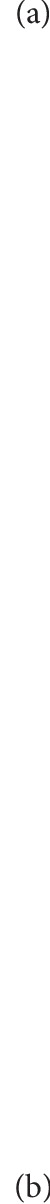
Verrucous Carcinoma. Marked digitated acanthosis of is associated with bulbous aggregates of squamous epithelium, producing a pushing border. Cytologic atypia was minimal. A focus of SCAP is present at the lower left 20x.

**Figure 3:**
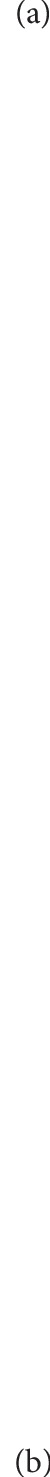
SCAP. Columnar epithelial cells with decapitation secretion line a papillated structure. A myoepithelial cell layer was present. The stroma contains numerous plasma cells 20x.

**Figure 4:**
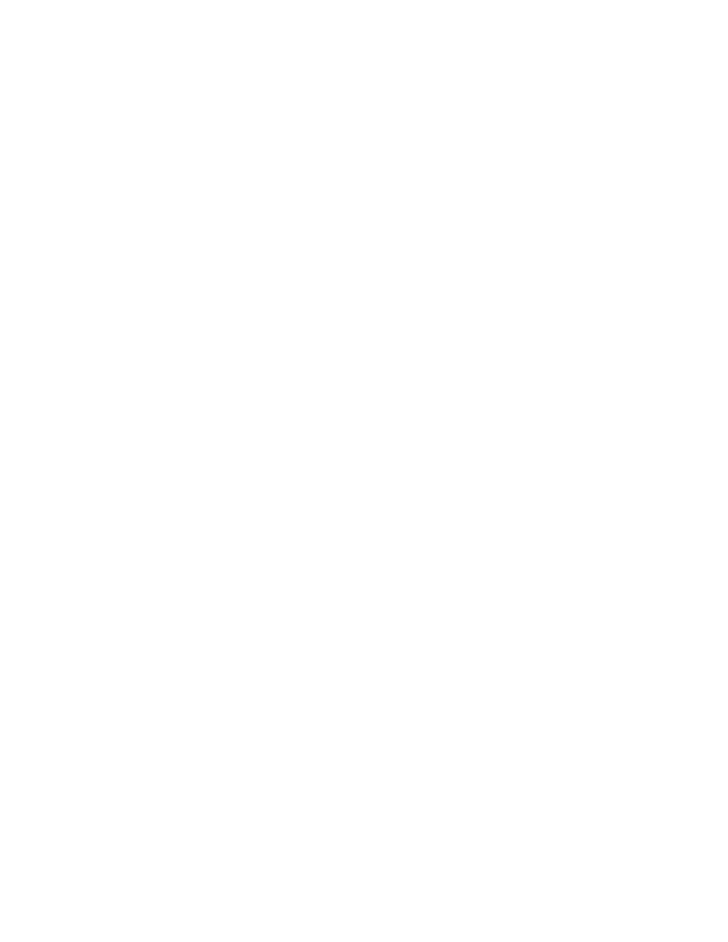
BCC. Aggregates of basaloid epithelial cells with peripheral palisading and central necrosis 200x.

On initial visit, a 6.0 cm deep shave excision is taken—showing transected verruca. On histopathologic examination, a large verrucous proliferation comprised of stratified squamous epithelium with slight atypia is seen (Figure 2). Focally, aggregates of atypical basaloid cells with peripheral palisading connect to the epidermis and extend into the dermis with some associated necrosis. In addition, there are foci of apocrine epithelial cells with a peripheral myoepithelial cell layer and associated stromal plasma cells. These findings show that a large verrucous proliferation is present along with an “incidental” syringocystadenoma papilliferum (Figure 3). Additionally, there is a focal associated nodular basal cell carcinoma (Figure 4). The lesion extended to the fragmented peripheral edges and deep base of the specimen. Clinicopathologically, it was determined that the deep and spreading nature of the verrucous proliferation alongside cellular atypia confirmed a diagnosis of verrucous carcinoma.

The patient underwent a wide radical excision of the scalp lesion with a full-thickness skin graft application harvested from the left lower abdomen. It was determined that the edges of the specimen showed no indication of any lesion remaining at the biopsy site.

## 3. Discussion

This case represents the fourth documented case of a coexisting SCAP and VC, but it is further complicated by a transformation to BCC. While there is a well-documented association between SCAP and transformation to BCC in 10% of cases, this case reports a collision of three diverse tumors.

Syringocystadenoma papilliferum is an uncommon benign adnexal tumor that can present as either a solitary papule, several papules in a linear arrangement, or as a plaque. Plaques are most closely associated with scalp lesions. A nevus sebaceous of Jadassohn is associated with up to one-third of SCAP [3]. There was no clinical evidence of a preexisting lesion, such as NSJ, in this patient. On histology, SCAP consists of a cystic and papillary growth of epithelial elements projecting downward into the dermis and opening onto the skin surface. Usually the tumor consists of two layers of epithelial cells: the peripheral layer is cuboidal and the inner, columnar cell layer shows evidence of decapitation secretion. The connective tissue stroma of the papillomatous projections is marked by rich infiltration of inflammatory cells; most notably there is a large number of plasma cells. It is believed that the origin of SCAP is from either eccrine or apocrine structures and their related undifferentiated pluripotent cells [5]. In addition to BCC, this benign tumor also has the potential to transform to metastatic adenocarcinoma or ductal carcinoma [3].

Verrucous carcinoma is an uncommon low-grade squamous cell carcinoma characterized clinically as a slowly but relentlessly enlarging warty tumor that has a very low metastatic potential—even if the tumor is large and has been present for many years. “Cutaneous” VC, also known as “papillomatosis cutis carcinoides,” is a rarer presentation in comparison to its variants more often reported on the genitalia, oral mucosa, and on the soles of the feet [4]. While the broad category of VC are histologically similar, the distinct etiology of VC can be dependent on its location. The most cited etiology is HPV, chronic irritation, and/or inflammation with the latter two being more often seen in oral VC—also known as an Ankerman tumor. Plantar warts and genital warts most often represent an HPV etiology of VC, resulting in an epithelioma cuniculatum and a Buschke–Loewenstein tumor, respectively. On histology, all types of VC represent a tumor of keratinocytes characterized by blunt projections of well-differentiated epithelium, supported by an edematous stroma with chronic inflammatory cells of lymphohistiocystic origin at its infiltrating margins. Bulbous islands of benign-appearing epithelium infiltrating the dermis represent an essential element of VC—local infiltration [4].

Surgical excision has been determined to be the most successful treatment for VC. Of note, VC can be particularly difficult to treat with radiotherapy—associated with rapid anaplastic transformation of the site despite regression of the tumor [4].

We present this interesting and unusual case to further illustrate the unlikely association of VC with a BCC-transformed SCAP. Previous reports examined an association of SCAP and VC located on the thigh and in the sacral region [1, 6, 7]. This case represents an expansion of this association to include the scalp as a possible site. Furthermore, this places emphasis on a possible chronic irritation etiology for VC. We believe that the SCAP may not be an incidental finding within the specimen but a possible underlying, initial compromise in this lesion.

## Abbreviations

SCAP: Syringocystadenoma papilliferum
BCC: Basal cell carcinoma
VC: Verrucous carcinoma
HPV: Human Papilloma Virus
NSJ: Nevus sebaceous of Jadassohn.
